# The impact of online education during the Covid-19 pandemic on the professional identity formation of medical students: A systematic scoping review

**DOI:** 10.1371/journal.pone.0296367

**Published:** 2024-01-05

**Authors:** Jonathan Zhen Liang, Donovan Kai Wei Ng, Vijayprasanth Raveendran, Mac Yu Kai Teo, Elaine Li Ying Quah, Keith Zi Yuan Chua, Jun Kiat Lua, Jasmine Lerk Juan Owyong, Andrew Vimal Vijayan, Nur Amira Binte Abdul Hamid, Ting Ting Yeoh, Eng Koon Ong, Gillian Li Gek Phua, Stephen Mason, Warren Fong, Crystal Lim, Natalie Woong, Simon Yew Kuang Ong, Lalit Kumar Radha Krishna

**Affiliations:** 1 Yong Loo Lin School of Medicine, National University Singapore, Singapore, Singapore; 2 Division of Supportive and Palliative Care, National Cancer Centre Singapore, Singapore; 3 Division of Cancer Education, National Cancer Centre Singapore, Singapore, Singapore; 4 Department of Pharmacy, National Cancer Centre Singapore, Singapore, Singapore; 5 Duke-NUS Medical School, Singapore, Singapore; 6 Assisi Hospice, Singapore, Singapore; 7 Lien Centre for Palliative Care, Duke-NUS Medical School, Singapore, Singapore; 8 Palliative Care Institute Liverpool, Academic Palliative & End of Life Care Centre, University of Liverpool, Liverpool, United Kingdom; 9 Department of Rheumatology and Immunology, Singapore General Hospital, Singapore, Singapore; 10 Medical Social Services, Singapore General Hospital, Singapore, Singapore; 11 Department of Internal Medicine, Singapore General Hospital, Singapore, Singapore; 12 Division of Medical Oncology, National Cancer Centre Singapore, Singapore, Singapore; 13 PalC, The Palliative Care Centre for Excellence in Research and Education, Singapore, Singapore; Tehran University of Medical Sciences, ISLAMIC REPUBLIC OF IRAN

## Abstract

Evolving individual, contextual, organizational, interactional and sociocultural factors have complicated efforts to shape the professional identity formation (PIF) of medical students or how they feel, act and think as professionals. However, an almost exclusive reliance on online learning during the COVID-19 pandemic offers a unique opportunity to study the elemental structures that shape PIF and the environmental factors nurturing it. We propose two independent Systematic Evidence-Based Approach guided systematic scoping reviews (SSR in SEBA)s to map accounts of online learning environment and netiquette that structure online programs. The data accrued was analysed using the clinically evidenced Krishna-Pisupati Model of Professional Identity Formation (KPM) to study the evolving concepts of professional identity. The results of each SSR in SEBA were evaluated separately with the themes and categories identified in the Split Approach combined to create richer and deeper ‘themes/categories’ using the Jigsaw Perspective. The ‘themes/categories’ from each review were combined using the Funnelling Process to create domains that guide the discussion. The ‘themes/categories’ identified from the 141 included full-text articles in the SSR in SEBA of online programs were the content and effects of online programs. The themes/categories identified from the 26 included articles in the SSR in SEBA of netiquette were guidelines, contributing factors, and implications. The Funnelling Process identified online programs (encapsulating the content, approach, structures and the support mechanisms); their effects; and PIF development that framed the domains guiding the discussion. This SSR in SEBA identifies the fundamental elements behind developing PIF including a structured program within a nurturing environment confined with netiquette-guided boundaries akin to a Community of Practice and the elemental aspect of a socialisation process within online programs. These findings ought to be applicable beyond online training and guide the design, support and assessment of efforts to nurture PIF.

## Introduction

Developing altruistic, ethical, humanistic and accountable physicians pivots on nurturing a medical student’s professional identity formation (PIF) [1, 2]. However, medical education continues to struggle to understand and shape how medical students feel, act and think as professionals [3]. Sarraf-Yazdi et al. [4] attribute current gaps in understanding PIF to a failure to understand the impact of environmental, organizational, educational, research, clinical, individual, psychosocial, and contextual factors on the PIF process.

The shift from in-person, multi-actor educational interactions to a pandemic-induced online medical education program, offered a unique opportunity to study the key influences shaping PIF [5]. The nature of online platforms creates physical boundaries between virtual and physical programs, oft-password controlled access, and structured approach access, shaping interactions and guiding progress, attenuating some of the many influences impacting learning. This allows the essential aspects shaping PIF to come to the fore [6]. With such insights likely to inform efforts to nurture PIF in any training situation in medical school and beyond [7], we ask the question “*how does an online education program shape a medical student’s PIF*?”.

## Theoretical framework

### The mentoring ecosystem

Conceiving online training programs as self-contained, structured programs with clear boundaries, and a distinct training trajectory for its multiple learners, tutors, and the host organization (henceforth stakeholders) draws similarities with the mentoring ecosystem [8]. The lens of the mentoring ecosystem focuses attention to structural and environmental facets that map a medical student’s progress. It also allows characterization of PIF through use of the Krishna-Pisupati Model for Professional Identity Formation (henceforth KPM).

The mentoring ecosystem pivots on the presence of clear boundaries that limit the effects of external influences on the progress of mentees along its structured, stage-based trajectory. This structured approach includes its specified learning objectives [9], goals [10, 11], timelines and professional standards [12, 13], codes of conduct, roles, responsibilities, expectations [14, 15], implicit norms [16], culture [17], artifacts, sociocultural norms and expectations and legal requirements [18–20] (henceforth *netiquette*); longitudinal mentoring support, stage based assessment program and its nurturing mentoring environment. These features liken the mentoring ecosystem to a Community of Practice (CoP) or *“persistent*, *sustaining social network of individuals who share and develop an overlapping knowledge base*, *set of beliefs*, *values*, *history and experiences focused on a common practice and/or mutual enterprise”* [21]. Current thinking suggests that CoPs are fundamental to PIF.

Concurrently within a structured program, the mentoring ecosystem’s spiral trajectory and longitudinal support and assessment processes supports the *Socialisation Process* or the process by which medical students are introduced and integrate new experiences. This *“process in which the characteristics*, *values*, *and norms of the medical profession are internalised*, *resulting in an individual thinking*, *acting and feeling like a physician”* is another critical aspect in nurturing PIF. The KPM captures evolving notions of PIF amidst maturing competencies and insights, shifts in belief systems, contextual considerations, and psycho-emotional states along the spiral mentoring trajectory [22–27].

### The Krishna-Pisupati Model of PIF

The KPM outlines adaptations to a medical student’s belief systems to create a context appropriate identity that is consistent with their current belief systems (*congruence*) and regnant social, organizational, and professional standards and beliefs (*social validation*) within a boundaried and structured program [6]. There are four aspects to an individual’s belief systems. These correspond to the Innate, Individual, Relational and Societal aspects of the individual’s self-concepts of identity or personhood depicted by the Ring Theory of Personhood (henceforth RToP) at the heart of the KPM [28–31] (S1 Fig).

When ‘life experiences’ are introduced and are integrated into the religious and cultural beliefs, moral values, and ethical principles in the Innate Ring; the beliefs system related to autonomous function and individual characteristics in the Individual Ring; the belief systems governing personal relationships are housed within the Relational Ring and/or the belief system guiding peripheral relationships and societal, professional, and legal expectations within the Societal Ring [29, 30, 32, 33], an *event* occurs. An *event* that is in sync with current belief systems creates *resonance*. *Synchrony* occurs when resonant aspects of the belief system are reprioritised to better address an *event*. When an *event* clashes with prevailing beliefs, dissonance arises. Dissonance in one ring is termed *disharmony*, whilst dissonance in two or more rings generates *dyssynchrony*.

*Sensitivity*, or detecting the presence of *resonance*, *synchrony*, *disharmony* and *dyssynchron*y, prompts medical students to evaluate the need for adaptations to their current belief systems (*judgement*) and determine their ability and readiness to make the change (*willingness*). To sustain their overall identity, and ensure *congruence* and *social validation*, the medical student must prioritise adaptations and their iterations of the *identity work* suits the settings, context, and practice *(balance)* [34]. It is suggested that evidence of *sensitivity*, *judgement*, *willingness*, *balance* and *identity work* points to development of PIF.

## Methodology

We carried out two independent systematic scoping reviews (SSR)s of netiquette and online environment. Focus on netiquette was informed by initial reviews showing significant overlap between structure and netiquette and that reviews of netiquette better captured accounts of codes of practice.

We adapted Krishna’s Systematic Evidence-Based Approach (SEBA) to guide the two SSRs (henceforth SSR in SEBA) [5, 8, 28, 30, 35–38]—the Dual-SEBA approach (S2 Fig). The Dual-SEBA’s constructivist approach [36, 39–44] and relativist lens [45–48] acknowledges belief systems, narratives, developing competencies, new life experiences, PIF, and netiquette as sociocultural constructs shaped by regnant environmental considerations, desired characteristics and expectations; and the medical student’s narratives, contextual factors, values, beliefs, and principles [49, 50].

Each stage of the Dual-SEBA approach was guided by an expert team which comprised of a librarian from the National University of Singapore’s (NUS) Yong Loo Lin School of Medicine (YLLSoM) and local educational experts and clinicians at YLLSoM, National Cancer Centre Singapore, Palliative Care Institute Liverpool, and Duke-NUS Medical School.

### Stage 1 of SEBA: Systematic approach

Each research team employed the PCC (Population/Concept/Context Study design) format and PRISMA checklist (see S1 File) to guide their primary research questions [51].

#### 1. Netiquette

With only a limited number of articles on the topic, the primary research question extended beyond the Covid-19 timeframe and focused on “*What is known about netiquette in online programs in medical schools*?” and the secondary research question was “*What are the features*, *causes and implications of lapses in netiquette in online programs in medical schools*?” (Table 1).

**Table 1:** PCC, inclusion and exclusion criteria applied to database search for netiquette.

PCC	Inclusion Criteria	Exclusion Criteria
**Population**	• Undergraduate and postgraduate medical students within clinical and/or medical settings	• Practicing physicians • Resident physicians, fellows • Teaching faculty, master’s programmes, Higher education programmes • Allied health specialities such as pharmacy, dietetics, chiropractic, midwifery, podiatry, speech therapy, occupational and physiotherapy • Non-medical specialities such as clinical and translational science, alternative and traditional medicine, veterinary, dentistry • Non-medical students
**Concept**	• Various standards of netiquette/ etiquette / professionalism in online learning / virtual environments set out by analysing: ○ Standards for virtual/ online meetings or tutorial netiquette/ etiquette / professionalism ○ Impact of standards used on virtual/ online meetings or tutorials ○ Infringement of standards in virtual/ online meetings or tutorials ○ Suggestions on how to facilitate a more conducive/optimal online learning experience ○ Assessing online professionalism and netiquette	• Virtual reality, virtual simulations, web-modules without interaction between tutors and students, videos, podcasts • Online patient education, web-based patient education, public education • Continuing medical education, professional development • Aspects of clinical research (disease, treatment, epidemiology) • Global health or public health
**Context**	• Virtual/online meetings or tutorials or video conferencing in the context of distance education	• Face-to-face education, didactic education, hands-on teaching, on-site teaching

Independent searches were conducted on PubMed, SCOPUS, ERIC, Google Scholar, Embase between 12^th^ September 2022 and 21^st^ January 2023 for articles published between 1^st^ January 2000 and 31^st^ December 2021 on online professionalism and standards of practice in online interactions within medical schools. The full search strategy is enclosed in the supplementary file (S2 File).

#### 2. Online medical training during Covid-19

To evaluate online medical training programs during the Covid-19 pandemic, the research and expert teams determined the primary research question to be *“What is known of online medical training programs during the Covid-19 pandemic*?*”*. The secondary research question was *“How are online medical training programs structured*, *assessed and supported during the Covid-19 pandemic*?*”* (Table 2).

**Table 2:** PCC, inclusion criteria and exclusion criteria applied to database search for online medical training during Covid-19.

PCC	Inclusion Criteria	Exclusion Criteria
**Population**	Undergraduate and postgraduate medical students within clinical and/or medical settings	• Practicing physicians • Resident physicians, fellows • Teaching faculty, master’s programmes, Higher education programmes • Allied health specialities such as pharmacy, dietetics, chiropractic, midwifery, podiatry, speech therapy, occupational and physiotherapy • Non-medical specialities such as clinical and translational science, alternative and traditional medicine, veterinary, dentistry • Non-medical students
**Concept**	• Program approaches, modalities, processes, objectives, motivations, challenges, facilitating characteristics/resources in supporting professionalism • Impact of supporting online professional identity formation on host organisation, assessors, and medical students and physicians ○ Professional identity formation outcomes such as on career choices (including academia positions/careers)	• Virtual reality, virtual simulations, web-modules without interaction between tutors and students, videos, podcasts • Online patient education, web-based patient education, public education • Continuing medical education, professional development • Aspects of clinical research (disease, treatment, epidemiology) • Global health or public health
**Context**	• Virtual/online meetings or tutorials or video conferencing in the context of distance education	• Face-to-face education, didactic education, hands-on teaching, on-site teaching

In surveying extant literature on online medical training programs during the Covid-19 pandemic, the second research team extended Stojan et al. [52]’s review on online learning developments in undergraduate medical education during Covid-19, beyond articles published on the MedEdPublish portal. Snowballing of relevant articles from the included articles was also proposed to ensure a comprehensive review and the inclusion of key articles.

Members of the research team conducted independent searches on PubMed, Embase, ERIC and Scopus between 17^th^ December 2022 and 17^th^ February 2023 for articles published between 1^st^ January 2019 to 31^st^ December 2022.

*Searching*. To ensure a sustainable review the expert teams limited the inclusion criteria in keeping with Pham et al. [53]’s approach to scoping reviews. Each team independently studied the database and discussed their findings, adopting Sandelowski and Barroso [54]’s ‘negotiated consensual validation’ to attain consensus on the final list of titles to be reviewed.

### Stage 2 of SEBA: Split approach

Krishna’s ‘Split Approach’ ensures that novel aspects of the area of interest are not omitted [39, 53–57]. For each review, two independent groups of researchers analysed the included articles concurrently using Braun and Clarke [58]’s approach to thematic analysis and Hsieh and Shannon [59]’s approach to directed content analysis.

Employing Braun and Clarke [58]’s approach to thematic analysis, the first team of researchers independently reviewed the included articles to map patterns in the data and synthesise a code book to code the remaining articles. Guided by an inductive approach, subthemes were reorganised into themes that best described the data [60]. ‘Negotiated consensual validation’ determined the final list of themes.

The second research team adopted Hsieh and Shannon [59]’s approach to directed content analysis, deriving codes from Ahmed et al. [61]’s review entitled *“Model for utilizing distance learning post COVID-19 using (PACT)™ a cross sectional qualitative study”* to encapsulate key aspects of online education programs and netiquette. In the presence of a working theory, Hsieh and Shannon [59]’s approach to directed content analysis promises to capture all evidence of phenomena identified in the KPM and attenuate concerns regarding the omission of negative findings and new considerations attributed to thematic analysis [4, 33, 62–64]. Hsieh and Shannon [59]’s approach to directed content analysis also provides ‘*supporting and un-supporting evidence for a theory*’ which in turn allows for KPM to be ‘*supported and extended*’ [59, 65, 66]. The deductive approach adopted allows confirmation, expansion, retesting and study of the KPM theory beyond the mentoring setting [66–68]. This approach acts as a check and balance [69] to reflexive thematic analysis that pivots on *coding reliability* and use of Cohen’s Kappa to assess the degree of consensus between researchers coding the same piece of data; *code books* that contain a shared understanding of the codes and themes; *reflexive thematic analysis* which recognises the role of researcher’s interpretation of the codes; and the employ of multiple researchers to ‘*sense check’* the data.

Here, the Split Approach is useful particularly when Cohen’s Kappa is not employed, given that coding is seen as part of a training process for new researchers. The presence of independent data from different sources also reduces concerns about the trustworthiness [70].

### Stage 3 of SEBA: Jigsaw perspective

Reimagined as pieces of a jigsaw puzzle, complementary elements of themes in each review were combined with the categories identified in direct content analysis to create bigger pieces of the puzzle or ‘themes/categories. This process was guided by Phases 4 to 6 of France et al. [71]’s approach to meta-ethnography.

### Stage 4 of SEBA: Funnelling process

France et al. [71]’s approach also guided the Funnelling Process which juxtaposed the themes/categories from each review to form domains.

## Results

### a. Online programs

**Fig 1:**
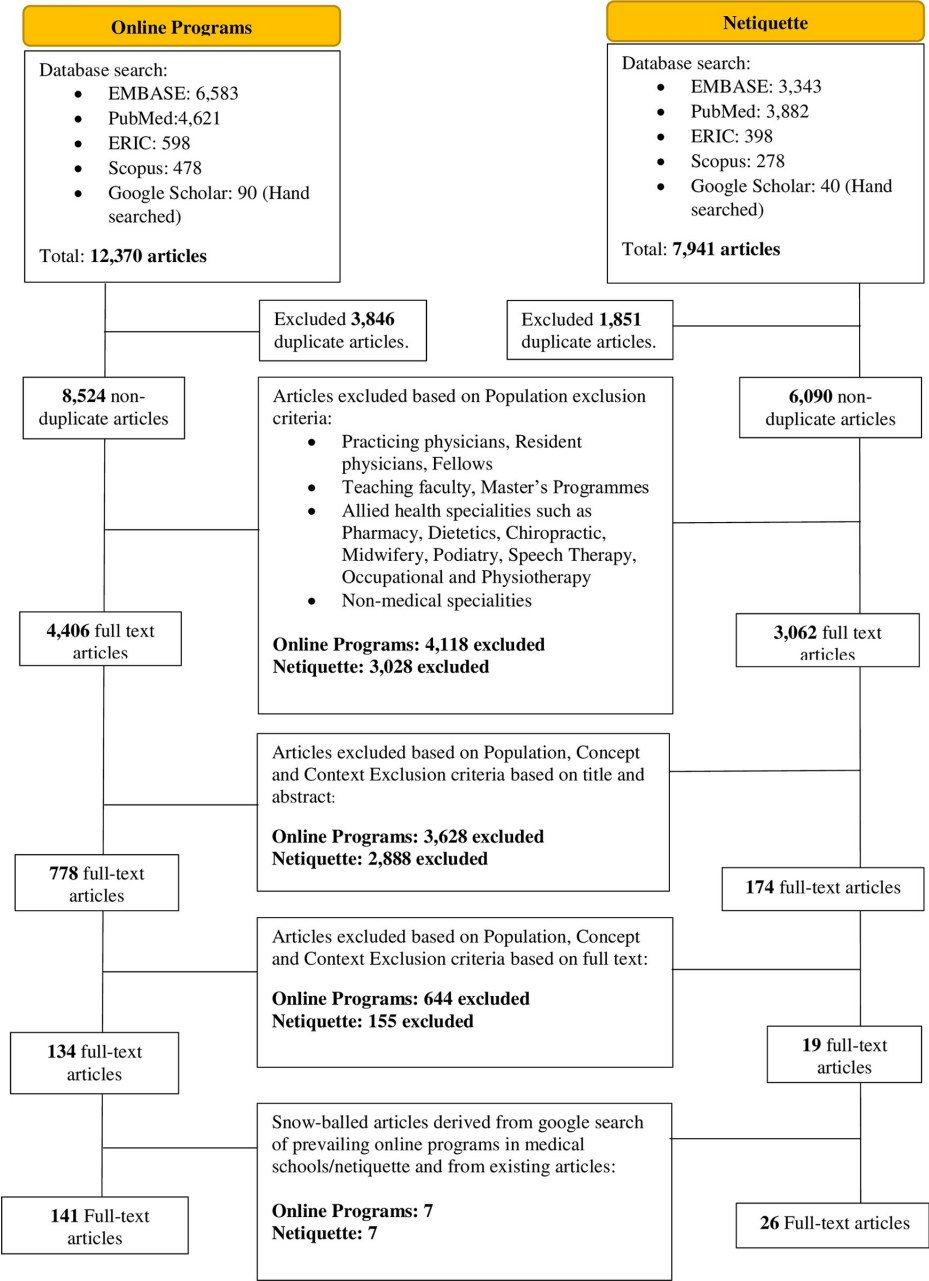
PRISMA flow chart for online programs and netiquette.

12370 abstracts were reviewed, 4406 full text articles were evaluated and 134 articles were included. With snowballing identifying seven articles, **141** full text articles were included (Fig 1). 65 were quantitative studies, five qualitative studies, two mixed studies, and 69 were descriptive/opinions/proceedings/reviews/perspectives/monographs. The Jigsaw Perspective identified two themes/categories—the content of current programs and effects of online programs.

### b. Netiquette

A total of 6115 abstracts were reviewed, 174 full text articles were evaluated, and 19 articles were initially included (Fig 1). Seven additional articles containing the existing netiquette and online professionalism guidelines of medical schools were snowballed from a Google search and from existing articles, yielding a total of **26** final included articles. Six were quantitative studies, two were qualitative studies and 18 were descriptive/opinions/proceedings/reviews/perspectives/monographs. The themes/categories identified were current guidelines, contributing factors, and their implications.

#### The iterative process of SEBA

With the initial findings suggesting the presence of features of CoPs and the Socialisation Process, Hsieh and Shannon [59]’s approach to directed content analysis was used to draw on codes and categories from current data on the KPM [66, 72, 73]. This process created two additional domains. The four domains were features of: 1) current programs; 2) netiquette, 3) CoP; and 4) KPM.

#### Domain 1. Features of current programs

Often replacing traditional approaches, the Covid-19 pandemic-induced curricula boosted support of medical students at all stages of their training and catered to the individual needs of medical students from different backgrounds, settings, expectations and different levels of knowledge, skills, and experience [5, 74–86]. Wooliscroft [87] and Alkhowailed et al. [88] suggested that the Covid-19 pandemic-induced curricula changes had cemented telemedicine, simulated learning, and extended reality learning’s role in modern medical education.

The content covered included the expansion of online content in emergency care [52], confidentiality, safety, awareness of online personas [89–91], and netiquette [89, 90]. In addition, it has enhanced access to ‘knowledge banks’ [92–96], and encouraged more sustainable [97–106], innovative [82, 107–113], rewarding [114], flexible [94, 115], context-appropriate [76, 105, 112, 116–120], engaging [76, 121], interactive [81, 122] and interprofessional educational approaches [123, 124].

These enhancements better aligned expectations, structuring, assessment, and support of online programs [85, 125], improved critical thinking, metacognitive and problem-based thinking; boosted engagement and teamwork; increased achievement of learning objectives [52, 105, 116, 118–120, 126, 127], access to learning [52, 94, 97, 128–130], and knowledge acquisition and satisfaction rates [131]. Gordon et al. [132], Daniel et al. [133], Dedeilia et al. [107], Stojan et al. [52] and Grafton-Clarke et al. [101] credited online programs with building confidence and skills, role modelling professional values [134], supporting reflective practice [135], and nurturing PIF. Rose [134], Aluri et al. [135], and Stetson et al. [136] reported that online interventions contextualised learning and provided users with authentic clinical experiences.

The approach to online teaching also impacted outcomes. Though asynchronous online sessions [93, 101, 106, 137, 138] offered convenient study [78, 94, 97, 104, 139–142] and fostered work-life balance [96, 123, 143–146], medical students preferred synchronous sessions [97, 104, 139]. Synchronous sessions countered social isolation [113, 117, 147], provided peer-mentoring and complemented face-to-face learning [93, 113, 147–149].

#### Domain 2. Features of netiquette

The ill-effects of online education were often not discussed in depth and are summarised in Table 3 for ease of review. These varied considerations underpin the need for structuring and policing of practice. It also helps shape the training trajectory.

**Table 3:** Possible contributing factors for reduced online professionalism and netiquette.

Theme	Possible Factor	References
Infrastructure	Lack of adequate, robust and accessible infrastructure including safe, stable internet connections and conducive learning spaces	[5, 75, 76, 86, 100, 101, 110, 112, 118, 141, 146, 148–189]
	Poor technical skills	[75, 101, 104, 110, 112, 118, 141, 148, 155, 165, 167–171, 177, 182, 183, 190–195]
	Lack of faculty training	[76, 139, 146, 155, 165, 173, 178, 180, 183, 192, 196]
	Lack of mentoring support	[146, 191–193, 197, 198]
	Inadequate assessment	[74, 88, 94, 113, 149, 178, 180, 194, 199–201]
	Lack of institutional support	[74, 76, 173, 180, 195, 202, 203]
Teaching Issues	Dissatisfaction with lessons due to methods of instruction (teaching style, lesson type, teaching pace)	[96, 110, 155, 156, 157, 161, 173, 182, 194, 199, 204–208]
	Lessons are too long	[110, 140, 155, 156, 177, 191, 209]
	Topics are too difficult	[210, 211]
	Limited exposure to specialist training	[100, 110, 142, 143, 165, 193, 199, 200, 205, 212–216]
	Lack of clinical exposure	[100, 105, 110, 140, 143, 165, 166, 196, 199, 203, 205, 214–222]
	Lack of exposure to unique patient groups as a result of limited hospital postings	[22, 197, 222, 223]
Time Management Issues	Poor scheduling/conflicts in scheduling	[150, 224, 225]
	Poor work-life balance	[180]
	Overall time commitment is too much	[141, 156, 197, 209]
Mental Health	Reduced motivation	[97, 104, 183, 184, 208, 226–229]
	Burnout	[104, 210, 230, 231]
	Stress (academic, psychosocial, familial) and anxiety	[125, 136, 141, 156, 147, 197, 206, 208, 215, 229, 232–241]
	Loneliness	[136, 206, 208, 220, 241]
	Mental health deterioration	[149, 197, 208, 226, 229, 231, 234, 242]
Behavioural Change	Cynicism	[178, 231, 243]
	Arrogance/irritation and frustration	[208]
	Distractions and reduced concentration	[97, 208, 220, 241, 244–248]
	Lack of self-discipline	[146, 195, 220, 243, 245]
	Lack of cultural sensitivity	[198]
Individual	Inadequate environment for partaking in online meetings	[141, 156, 177, 180, 226]
	Failure to turn on/ turning off videos during discussion	[191, 247, 249, 250]
	Lack of attention	[77, 161, 251, 252]
	Lack of effective participation/ increased disengagement	[37, 93, 95, 142, 149, 180, 183, 191, 195, 216, 249, 253]
	Perceived lack of impact on learning	[88, 146, 163, 182, 196, 220]
	Differentiate personal and professional online identities	[7, 179, 181]
	Lack of a common understanding of expectations and codes of conduct	[181, 254]
Nature of Online Platforms	Inability to read non-verbal cues	[52, 177]
	Intrusion of privacy	[255]
	Health issues from viewing laptops and computer	[158, 256]
	Students are unaware of when to be professional	[255]
	No control of online profile	[161, 257]

However, differences in focus, duration, subject matter, level of sophistication, structure, assessment processes, and support and oversight of the program and participants across regnant online curricula, along with time and manpower constraints caused by the sudden shift to online education created differences in the content of published netiquette guidelines [89, 90]. The context specific nature of netiquette is summarised in Table 4 for ease of review.

**Table 4:** Content of netiquette guidelines.

Topics	Specific Guidelines	References
Safety	Institutional login or reporting attendance	[168, 258, 259]
	Warm calls to tell students that they will be asked questions	[105]
	Allow time to respond	[105]
	Keep personal passwords private; use own credentials	[260, 261]
	Establish clear expectations and codes of practice	[105, 262, 263]
Awareness of Online Persona	Professional backgrounds for video meetings	[264, 265]
	Standardise format of names	[266]
	Punctuality	[267]
	Quiet and private workspace. Use headphones to ensure privacy	[264–267]
	Stable internet connection	[264]
	Keeping camera switched on	[90, 249, 266–268]
	Uploading photo as a display picture	[90, 269]
	Appropriate attire	[255, 265–267, 270]
	Mobile usage is not permitted if it compromises patient care and privacy	[265]
	Use of the chat function to ask questions to avoid disruption	[259, 271, 272]
	Ask specific questions	[188]
	Communicate with respect, sensitivity and professionally	[260, 261, 273, 274]
	Communicate with respect	[52, 188]
	Do not attach unnecessary files	[273]
Confidentiality	No recording without explicit consent from all participants	[265]
	Do not copy a message or attachment without permission	[273]
	Provision of personalised and private feedback	[52, 99]
	Do not disclose patient identity, patient data, or patient images	[274, 275]

#### Domain 3. Features of a CoP

The physical separation between online and physical practice, the online approach, netiquette and structure of the program created boundaried programs. The program structures also advanced clear step-wise inculcation of knowledge, avenues to practice skills and an opportunity to introduce and integrate the values, beliefs, principles and attitudes espoused by the program. These structures served to gradually empower the medical students and give them more significant roles in the program reminiscent of the move from legitimate peripheral participation to key roles at the core of a CoP.

To validate the premise that online programs function like mentoring ecosystems or a modified CoP, the expert and research teams sought to identify features of CoPs drawn from Cruess et al. [276], Clement, Brown [277], Sherbino, Snell [278], Hean, Anderson [279], Hägg-Martinell, Hult [280], Buckley, Steinert [281] and de Carvalho-Filho, Tio [282] in accounts of online programs.

Whilst there was evidence of a ‘*persistent*, *sustaining social network’* and a*‘social network of individuals’;* evidence for *‘an overlapping knowledge base*, *set of beliefs*, *values*, *history and experiences’* could only be inferred [96, 110, 155, 156, 158, 161, 173, 182, 194, 199, 204–207]. Here, shared values, culture, goals, a common identity and a welcoming environment were drawn from accounts of online programs seeking to engage and challenge medical students, set and align expectations, and nurture a conducive learning environment [150, 224, 225].

Similarly, evidence for a structured and guided approach, and flexible and adaptive support mechanisms were implied from the presence of a discrete online program confined by clear physical boundaries, supplemented by standards, netiquette, formal curriculum, learning objectives and a learning trajectory (henceforth *structured online program*) [167–169].

Concurrently, data on the presence of a learning trajectory, akin to the notion of a mentoring trajectory, that guides progress is deduced from accounts of achievement of learning objectives [52, 105, 116, 118–120, 126, 127], alignment of expectations, structuring, assessment, and support of online programs [85, 125], and contextualised learning within authentic clinical experiences [167, 168, 169].

Structure was also evident from the provision of longitudinal role modelling of professional values [134], supervision, feedback and mentoring to accommodate the learner’s individual goals and needs and support of reflective practice [135]. Efforts to foster work-life balance [96, 123, 143–146], counter social isolation [113, 117, 147], complement face-to-face learning [93, 113, 147–149] and in nurturing PIF [97, 208, 220, 241, 244–248] hint at the presence of a flexible, personalised, responsive, assessment driven approach.

These features, however, were not consistent across the programs and the netiquette guidelines as evidenced by Tables 3 and 4.

#### Domain 4. Features of KPM

The impact of a structured online program on PIF on belief systems and identity is inferred. However, Stouffer et al. [283]’s account of a short week-long online arts and humanities course for second, third- and fourth-year medical students at John Hopkins University does merit attention. Here, the authors suggest that this intervention inspired the “process of psychological and social development that occurs within the larger context of overall identity formation” [296]. Other accounts also infer as much. Stojan et al. [52], Dedeilia et al. [107] and Grafton-Clarke et al. [111], for example, report that online learning enhanced cognitive capabilities, and facilitated greater engagement suggesting changes in the Individual and Societal Rings of the RToP [105, 114, 116, 118–120, 122, 127]. Other accounts revealed online programs encouraged medical students to become ‘change agents’ and actively reshape the education landscape [97, 113, 284–286]. Changes to teamwork [287], practice, thinking [131] and wellbeing also imply influence upon *sensitivity*, *judgement*, *willingness*, *balance*, *identity work* and *reflections* within the KPM.

Conversely, disrupted and ineffective learning [105, 158, 249, 250, 288–291], and a failure to meet learning objectives [289, 292] resulted in disharmony in the Individual Ring. There were also accounts of disharmony in the Relational Ring caused by poor tutor-learner relationships [61, 247, 252, 253, 269], reduced peer interactions [150, 199, 253] and increased isolation [206, 253, 293, 294]. Disharmony in these rings cascaded into dyssynchrony across the Societal, Relational and Individual Rings exaggerated by gaps in knowledge, skills, and attitudes [251], and poor interprofessional practice [142, 206, 293, 295, 296]. Overall, when unsupported, such dissonance culminated in ineffectual adaptations, further indicating wider impact upon *sensitivity*, *judgement*, *willingness*, *balance*, *identity work* and *reflections* within the KPM [293]. The effects [135, 220] of netiquette and online programs on the rings of the RToP within the KPM are summarised in Table 5.

**Table 5:** Effects of netiquette and online programs on RToP.

Positive	Negative
**Innate Ring**	
*“We were able to support science and the population, even before finishing our own studies. This is a strong feeling of usefulness, which was not there beforehand.” [220]*	*“What is a good life for my patient is what my patient wishes for that life to be*. *And I say that*… *to emphasize each patient’s individuality and to de-emphasize my ability to surmise what their view of “good” and “life’ and a “good life” might be*.*” (early-course cohort*, *essay 3)* *“For a long time, I equated a good life with the perfect life. I had a plan with specific goals regarding how I wanted my life to look… There was very little time spent in the present and appreciating what was actually going on whether positive or negative. I realized that the perfect life wasn’t all that good… Consequentially, I have made a lot of substantial changes to how I approach many aspects of my life, especially with regards to taking steps to stop and just be present in whatever moment I am currently experiencing…” (late-course cohort, essay 5) [135]*
**Individual Ring**	
*“A desire to partake in the management of the COVID crisis–in the end that’s why I became a doctor. An incredible learning opportunity.” [220]* *“Since e-learning has launched, we can have the professors’ words and lessons recorded, unlike in the past. This has allowed me to play it back and review it so I can analyze and interpret it better. I think it brought me deeper learning.” [150]* *‘Like keeping it completely separate. . . I have a lot of people I know in the course; I don’t have very many medical friends on Facebook because I want to keep it completely separate and people can’t find me because. . . I know that can affect your career later, so part of me wants to quit [Facebook] anyway.’ [255]*	*“I realized that my current training takes an important part of my life, and when it is altered, it is hard to find a work balance and the motivation to go on, the latter being also driven through group learning or clinical activities.” [220]* *“I was [. . .] less stimulated, I couldn’t directly ask questions to colleagues or tutors.” [220]* *“As long as it is not summative, we tend to do the minimum necessary.” [220]* *“The problem is that not all courses can be taught virtually, for example, history taking, physical exam, and bedside teaching cannot be done virtually.” [150]* *“Working from home limits my motivation. I procrastinate much more, which rapidly throws me into a vicious circle of stress and working to catch up: I don’t manage to motivate myself to study, causing me to get stressed and freeze, which again hinders me to work.” [220]* *“I wasn’t as diligent in my studying and the knowledge is clearly not acquired.” [220]* *“It was a highly enriching experience, but it has probably brought along large gaps in my training. Formative exams, the cancellation of all classwork [. . .] have caused knowledge gaps that will be quite hard to fill and we did not get many tools to over- come them. I think there will be groups that will be less well trained within the same cohort.” [220]*
**Relational Ring**	
*“Take time for myself, rest and enjoy my loved ones even more.” [220]* *“I feel confirmed in my ideas and desire to not neglect time with my family and my loved-ones.” [220]*	*“No link with other students at my place (I usually work by myself but always in contact with other students for questions about the learning objectives, organization,. . .).” [220]* *“The feeling of being left behind was quite strong, which was what had mostly changed compared to “normal” times.” [220]* *“The professors often upload offline sessions which are one-sided, and we cannot actively participate in practical terms. The professor puts a voice-over on slides and sends them. This cannot be like classes where you can raise your problems and ask questions. Therefore, students’ participation and inter- action are not seen in my point of view, and that is a big problem.” [150]* *This [Houseparty] restores a little of what is otherwise lost, so if something funny happens in the lecture, something funny is said, then we laugh about it together and so we could laugh or make a comment together. (4.2 S) [141]* *“My family did not realize that I am seriously busy in learning through online system and that put a lot of pressure on me.” [177]*
**Societal Ring**	
*“I had the impression that the medical students could be helpful, even bachelor students, that were able to take an active role in the hospital (incredible).” [220]* *“I discovered a new interest for family medicine and for people in precarious situations.” [220]* *“The feeling of belonging to the health care workers and the vision of their commitment.” [220]* *“I realize the importance of solidarity among health care professionals.” [220]* *“You get to give people back one of the most central elements of themselves, their mind… I find it difficult to think of a more fulfilling pursuit… My dream is to sustainably assist my patients as they pursue their own good life.” (late-course cohort, essay 2) [135]*	*‘I don’t know what professionalism means in the context of a university student. . .’ [255]* *‘I’ve got a friend. . . she’s a doctor now and often I notice her [Facebook] status is [about] things like ‘‘So-and-so is sick of intravenous drug users” and I’m thinking, ‘‘This is awful; you can’t put that on Facebook” and I mean she’s not like naming names so maybe she thinks it’s ok. . . it’s always to do with something that’s happened at work.’ [255]*

#### Stage 5 of SEBA: Analysis of evidence-based and non-data driven literature

The inclusion of non-data-based articles such as position, perspective, commentaries, conference, reflective and opinion papers, editorials, oral presentations, letters, posters, forum discussions, blogs, interviews, surveys, governmental reports and policy statements from PubMed, Embase, SCOPUS, ERIC and Google Scholar raised concerns over biases in the analysis. To allay these concerns, the research team compared the themes elicited from data-driven publications with those from non-data-based articles. Similarities between the two groups assuaged concerns of biases.

## Discussion

### Stage 6 of SEBA: Synthesis of discussion

The “Best Evidence Medical Education (BEME) Collaboration Guide” [297] and the “Structured approach to the Reporting In healthcare education of Evidence Synthesis (STORIES)” [298] were used in the synthesis of responses to our primary research questions.

This Dual-SEBA review reveals online programs are comparable to mentoring ecosystems and capable of influencing a medical student’s narratives, their developing competencies, and their conduct and evolving PIF. In nurturing the “*transformative journey through which [a medical student] integrates the knowledge*, *skills*, *values*, *and behaviours of a competent*, *humanistic physician with [their] unique identity and core values*”, our Dual-SEBA approach addresses our overarching research objective of characterising the basic features required to support PIF in a structured program [299–302]. These include a structured curriculum; an established netiquette; an alignment of expectations; a consistent yet flexible approach; a clearly delineated learning trajectory; longitudinal support; a longitudinal assessment process; personalised and appropriate feedback; and a nurturing learning environment. These findings serve as a template for the design, support, and assessment of future programs in medical education and may be extrapolated to programs in different training settings and even beyond the medical student population.

Indeed, viewing PIF in online training programs as a series of interventions capable of shaping adaptations to belief systems, influencing identity work and asking questions of self-concepts of professional identity highlights several considerations. Whilst structure, a consistent approach, and a nurturing environment are pivotal, there must also be adequate acknowledgment of the individual needs of the participant population. Different narratives, belief systems, contextual considerations, abilities, levels of self-awareness and reflective capabilities undergird the need for a personalised support mechanism to run in tandem with a consistent training approach that seeks to cater for the needs of the general participant population. This also underscores the need for personalised, appropriate, specific, and timely assessments and mentoring support. Such support is essential to shaping a medical student’s *sensitivity*, *judgement*, *willingness*, *balance*, *identity work* and *reflections* and thus their belief systems, self-concepts of personhood and identity. Accessible personalised support is also pertinent when the ramifications of reflections may occur sometime after the experience and when evidence suggests that their effects impact all aspects of personhood and identity (Table 5). Indeed, this Dual-SEBA highlights the potential hazards of unsupported training in Table 3.

These findings underline the host organization’s role in ensuring effective design [4], oversight and support [5, 303] of the program and supporting faculty training programs and interprofessional education in online programs. Here, the absence of ‘train the trainers’ programs, vis-à-vis holistic assessments and longitudinal evaluations of the education program, is concerning. A further worry is the lack of consideration for communication platforms for accessible support and feedback and indeed the protected time afforded to faculty to meet the individual needs of their student population. Missing too are accounts of the long-term impact of online programs on PIF, oversight and program evaluations that will further guide structure and oversight of online programs.

## Limitations

Netiquette in medical education is a relatively under-reviewed and novel area in the existing literature. Gaps in current thinking are accentuated by our focus on the impact of online learning during Covid-19 on the PIF of only medical students.

Including articles in or translated into English may have also restricted the search results. With mainly North American and European-drawn data, these findings may not be as easily applicable beyond these regions.

## Conclusion

The insights provided in this Dual-SEBA highlights a number of new considerations that require evaluation. The importance of assessing this longitudinal and holistic developmental process suggests the need for more effective assessment tools, appraisal of the learning environment, training programs for trainers and portfolio use. Similarly, the involvement of interprofessional educational initiatives and potential assessments and support mechanisms also require further study. In light of the flexibility within the online program structure and the potential for cascading effects in PIF, we will focus our immediate attention on creating adaptive and longitudinal assessments of PIF as we look forward to engaging in this exciting field of medical education.

## Supporting information

S1 Fig Krishna-Pisupati Model of PIF. (TIF)

S2 Fig The Dual-SEBA approach. (TIF)

S1 File PRISMA checklist. (PDF)

S2 File Full search strategy. (DOCX)
