# Supplementary figures and images for: The impact of online education during the Covid-19 pandemic on the professional identity formation of medical students: A systematic scoping review
Source: PLoS One. 2024 Jan 5;19(1):e0296367. doi: 10.1371/journal.pone.0296367 (PMC10769105; doi:10.1371/journal.pone.0296367)

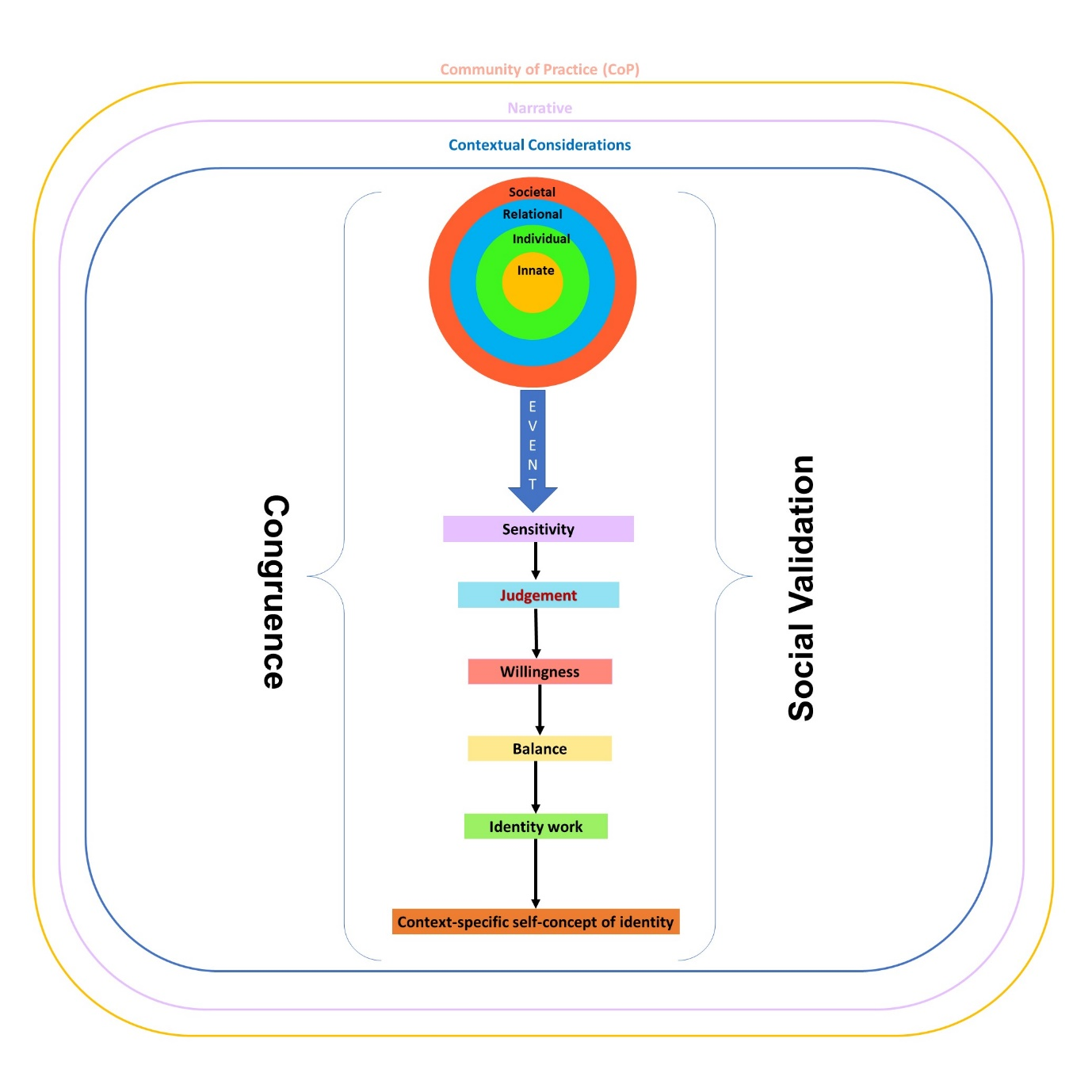

Supplement: S1 Fig — (TIF) [file pone.0296367.s001.tif]

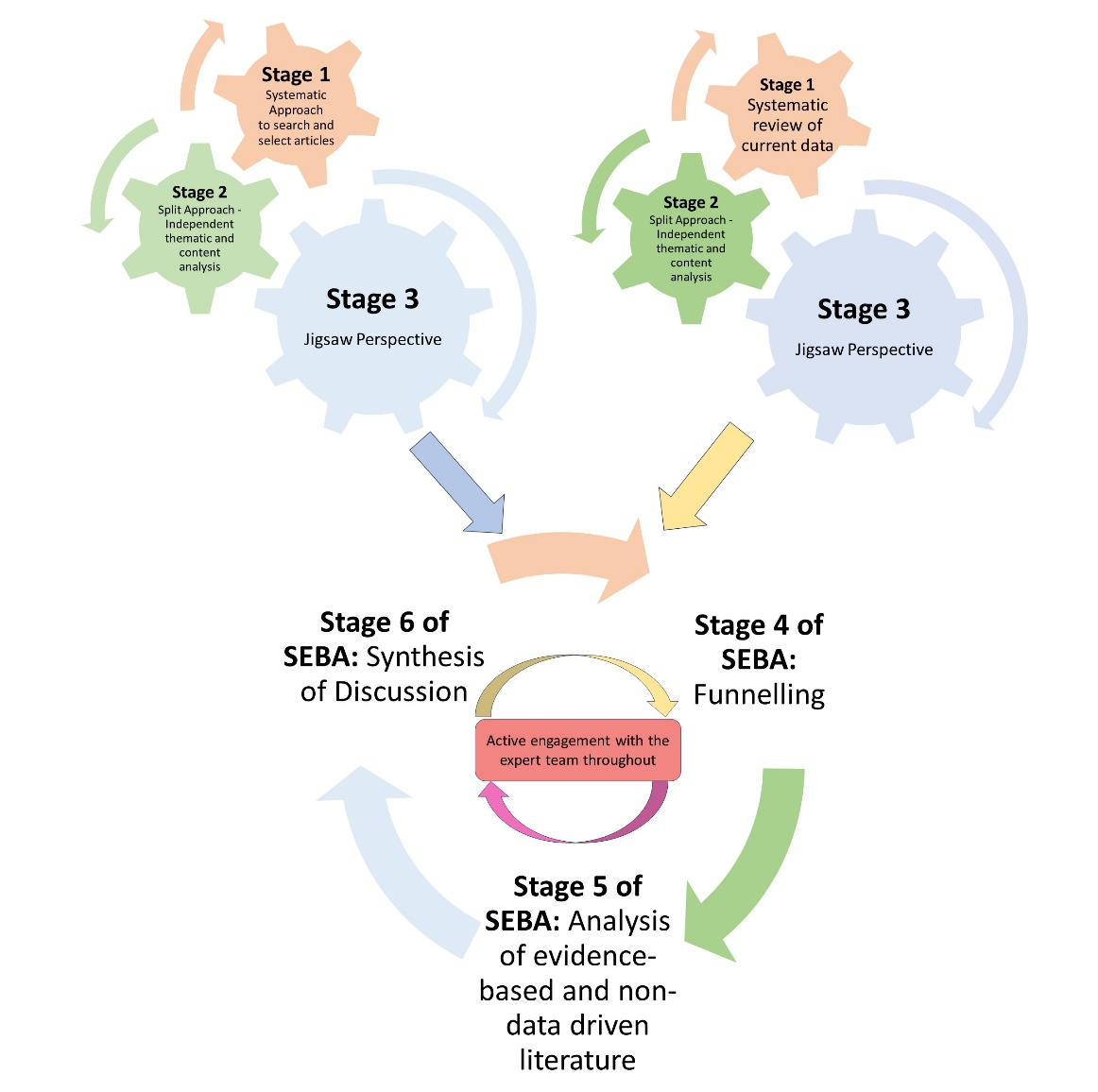

Supplement: S2 Fig — (TIF) [file pone.0296367.s002.tif]
