# Supplementary material for: The impact of online education during the Covid-19 pandemic on the professional identity formation of medical students: A systematic scoping review
Source: PLoS One. 2024 Jan 5;19(1):e0296367. doi: 10.1371/journal.pone.0296367 (PMC10769105; doi:10.1371/journal.pone.0296367)
Supplement: S2 File — (DOCX) [file pone.0296367.s004.docx]

**S2 File. Full Search Strategy**

***What is known about netiquette in online programs in medical schools*?”.**

PubMed

| 1 | "students, medical"[MeSH Terms] OR "medical student"[Title/Abstract] OR "medical students"[Title/Abstract] OR "medical undergraduate students"[Title/Abstract] OR "schools, medical"[MeSH Terms] OR "medical school*"[Title/Abstract]) AND ((2000/1/1:2022/12/31[pdat]) AND (english[Filter])) | 67664 |
| --- | --- | --- |
| 2 | ("education, distance"[MeSH Terms] OR "online education"[Title/Abstract] OR "online learning"[Title/Abstract] OR "online mentoring"[Title/Abstract] OR "e-learning"[Title/Abstract] OR "e-education"[Title/Abstract] OR "e-mentoring"[Title/Abstract] OR "electronic learning"[Title/Abstract] OR "electronic mentoring"[Title/Abstract] OR "electronic education"[Title/Abstract] OR "online program*"[Title/Abstract] OR “online training”[ Title/Abstract]) AND ((2000/1/1:2022/12/31[pdat]) AND (english[Filter])) | 15208 |
| 3 | ("Netiquette"[Title/Abstract] OR "online etiquette"[Title/Abstract] OR "e-etiquette"[Title/Abstract] OR "etiquette"[Title/Abstract] OR "Interprofessional Relations"[MeSH Terms]) AND ((2000/1/1:2022/12/31[pdat]) AND (english[Filter])) | 43604 |
|  | #1 AND #2 AND #3 | 32 |

Embase

| 1 | ("medical student" OR “medical school”)/exp OR (("medical student*" OR "medical undergraduate student*" OR “medical school*”):ab,ti) AND [2000-2022]/py AND [embase]/lim NOT ([embase]/lim AND [medline]/lim) AND [english]/lim | 50051 |
| --- | --- | --- |
| 2 | “distance learning”/exp OR ("online education" OR "online learning” OR "online mentoring" OR "e-learning" OR "e-education" OR "e-mentoring" OR "electronic learning" OR "electronic mentoring" OR "electronic education" OR "online program*" OR “online training”):ti,ab AND [2000-2022]/py AND [embase]/lim NOT ([embase]/lim AND [medline]/lim) AND [english]/lim | 7721 |
| 3 | “professional ethics”/exp OR ("Netiquette" OR "online etiquette" OR "e-etiquette" OR "etiquette"):ti,ab AND [2000-2022]/py AND [embase]/lim NOT ([embase]/lim AND [medline]/lim) AND [english]/lim | 341 |
|  | #1 AND #2 AND #3 | 2 |

Scopus

| 1 | TITLE-ABS-KEY ( "medical student*” OR "medical undergraduate students" OR "medical school”) AND ( PUBYEAR > 1999 ) AND ( PUBYEAR < 2023 ) AND LANGUAGE ( english ) | 109729 |
| --- | --- | --- |
| 2 | TITLE-ABS-KEY ( "e-learning” OR “elearning” OR “distance learning” OR “electronic learning” OR “online learning” OR “e-mentoring” OR “online program” OR “online training” OR “electronic education” OR “e-education” ) AND ( PUBYEAR > 1999 ) AND ( PUBYEAR < 2023 ) AND LANGUAGE ( english ) | 146711 |
| 3 | TITLE-ABS-KEY ( "etiquette” OR “netiquette” OR “net etiquette” OR “internet etiquette” OR “online etiquette” OR “e-etiquette”) AND ( PUBYEAR > 1999 ) AND ( PUBYEAR < 2023 ) AND LANGUAGE ( english ) | 2308 |
|  | #1 AND #2 AND #3 | 2 |

ERIC

| 1 | (MAINSUBJECT.EXACT.EXPLODE("Medical Students") OR MAINSUBJECT.EXACT.EXPLODE("Medical Schools")) OR title("medical student*" OR "medical undergraduate students" OR "medical school") OR abstract("medical student*" OR "medical undergraduate students" OR "medical school") | 3681 |
| --- | --- | --- |
| 2 | MAINSUBJECT.EXACT("Electronic Learning") OR title("e-learning" OR "elearning" OR "distance learning" OR "electronic learning" OR "online learning" OR "e-mentoring" OR "online program" OR "online training" OR "electronic education" OR "e-education") OR abstract("e-learning" OR "elearning" OR "distance learning" OR "electronic learning" OR "online learning" OR "e-mentoring" OR "online program" OR "online training" OR "electronic education" OR "e-education") | 24594 |
| 3 | abstract("etiquette" OR "netiquette" OR "net etiquette" OR "internet etiquette" OR "online etiquette" OR "e-etiquette") OR title("etiquette" OR "netiquette" OR "net etiquette" OR "internet etiquette" OR "online etiquette" OR "e-etiquette") | 172 |
|  | #1 AND #2 AND #3 | 0 |

***What is known of online medical training programs during the Covid-19 pandemic?***

PubMed

| 1 | "students, medical"[MeSH Terms] OR "medical student"[Title/Abstract] OR "medical students"[Title/Abstract] OR "medical undergraduate students"[Title/Abstract] OR "schools, medical"[MeSH Terms] OR "medical school*"[Title/Abstract] AND ((2000/1/1:2022/12/31[pdat]) AND (english[Filter])) | 67664 |
| --- | --- | --- |
| 2 | ("education, distance"[MeSH Terms] OR "online education"[Title/Abstract] OR "online learning"[Title/Abstract] OR "online mentoring"[Title/Abstract] OR "e-learning"[Title/Abstract] OR "e-education"[Title/Abstract] OR "e-mentoring"[Title/Abstract] OR "electronic learning"[Title/Abstract] OR "electronic mentoring"[Title/Abstract] OR "electronic education"[Title/Abstract] OR "online program*"[Title/Abstract] OR “online training”[Title/Abstract]) AND ((2000/1/1:2022/12/31[pdat]) AND (english[Filter])) | 15208 |
| 3 | ("COVID-19"[MeSH Terms] OR "covid*"[Title/Abstract] OR "coronavirus-19"[Title/Abstract]) AND ((2000/1/1:2022/12/31[pdat]) AND (english[Filter])) | 308767 |
|  | #1 AND #2 AND #3 | 1,680 |

Embase

| 1 | ("medical student" OR “medical school”)/exp OR (("medical student*" OR "medical undergraduate student*" OR “medical school*”):ab,ti) AND [2000-2022]/py AND [embase]/lim NOT ([embase]/lim AND [medline]/lim) AND [english]/lim | 50051 |
| --- | --- | --- |
| 2 | “distance learning”/exp OR ("online education" OR "online learning” OR "online mentoring" OR "e-learning" OR "e-education" OR "e-mentoring" OR "electronic learning" OR "electronic mentoring" OR "electronic education" OR "online program*" OR “online training”):ti,ab AND [2000-2022]/py AND [embase]/lim NOT ([embase]/lim AND [medline]/lim) AND [english]/lim | 7721 |
| 3 | “coronavirus disease 2019”/exp OR ("covid*” OR “coronavirus 19” or “coronavirus disease 2019”):ti,ab AND [2000-2022]/py AND [embase]/lim NOT ([embase]/lim AND [medline]/lim) AND [english]/lim | 130469 |
|  | #1 AND #2 AND #3 | 387 |

Scopus

| 1 | TITLE-ABS-KEY ( "medical student*” OR "medical undergraduate students" OR "medical school”) AND ( PUBYEAR > 1999 ) AND ( PUBYEAR < 2023 ) AND LANGUAGE ( english ) | 109729 |
| --- | --- | --- |
| 2 | TITLE-ABS-KEY ( "e-learning” OR “elearning” OR “distance learning” OR “electronic learning” OR “online learning” OR “e-mentoring” OR “online program” OR “online training” OR “electronic edication” OR “e-education” ) AND ( PUBYEAR > 1999 ) AND ( PUBYEAR < 2023 ) AND LANGUAGE ( english ) | 146711 |
| 3 | TITLE-ABS-KEY ( "covid-19” OR “coronavirus” OR “covid”) AND ( PUBYEAR > 1999 ) AND ( PUBYEAR < 2023 ) AND LANGUAGE ( english ) | 452329 |
|  | #1 AND #2 AND #3 | 915 |

ERIC

| 1 | (MAINSUBJECT.EXACT.EXPLODE("Medical Students") OR MAINSUBJECT.EXACT.EXPLODE("Medical Schools")) OR title("medical student*" OR "medical undergraduate students" OR "medical school") OR abstract("medical student*" OR "medical undergraduate students" OR "medical school") | 3681 |
| --- | --- | --- |
| 2 | MAINSUBJECT.EXACT("Electronic Learning") OR title("e-learning" OR "elearning" OR "distance learning" OR "electronic learning" OR "online learning" OR "e-mentoring" OR "online program" OR "online training" OR "electronic education" OR "e-education") OR abstract("e-learning" OR "elearning" OR "distance learning" OR "electronic learning" OR "online learning" OR "e-mentoring" OR "online program" OR "online training" OR "electronic education" OR "e-education") | 24594 |
| 3 | MAINSUBJECT.EXACT("COVID-19") AND abstract("covid-19" OR "coronavirus" OR "covid") AND title("covid-19" OR "coronavirus" OR "covid") | 3334 |
|  | #1 AND #2 AND #3 | 16 |
